# Integrating 3D bioprinting and neural stem cell therapy for central nervous system repair: implications for regenerative neurosurgery

**DOI:** 10.3389/fbioe.2026.1763855

**Published:** 2026-03-30

**Authors:** Yupeng Guo, Xuanwei Dong, Min Liu, Dongsheng Liu, Jianxin Wang

**Affiliations:** Department of Neurosurgery, Aviation General Hospital, Beijing, China

**Keywords:** 3D bioprinting, bioink, neural stem cells, neurodegenerative diseases, regenerative neurosurgery

## Abstract

The limited regenerative capacity of the central nervous system (CNS)poses a major challenge in neurosurgical interventions for spinal cord injuries, neurodegenerative diseases,and traumatic brain injuries. Traditional approaches offer structural stabilization but rarely achieve true neural tissue regeneration. Recent advances in regenerative medicine have spotlighted two promising strategies: 3D bioprinting and neural stem cell (NSC) therapy.3D bioprinting enables the fabrication of anatomically precise, biocompatible scaffolds that mimic native neural architecture, offering structural and biochemical support for tissue repair. Concurrently, NSCs capable of differentiating into neurons, astrocytes, and oligodendrocytes promote neurogenesis, synaptic integration,and immune modulation, making them attractive candidates for functional recovery. Despite extensive preclinical success, clinical translation of neural stem cell therapy remains inconsistent due to poor cell survival, uncontrolled differentiation, limited graft integration, and hostile post-injury microenvironments. This review critically examines why these limitations persist and argues that 3D bioprinting is not merely complementary, but essential for overcoming fundamental barriers to effective CNS regeneration. This review critically examines the convergence of 3D bioprinting and NSC therapy in neurosurgical applications. It discusses the design of bioinks, scaffold fabrication techniques,and the role of conductive and ECM-derived materials in supporting NSC viability and differentiation. The manuscript also explores preclinical and early-phase clinical trials (2015–2025), highlighting the therapeutic potential of NSC-loaded bioprinted constructs in spinal cord injury, Parkinson’s disease,and stroke. Further, we analyze the biophysical and biochemical cues within scaffolds that shape NSC fate and describe how 3D bioprinted models are being used for disease modeling and drug screening. This review synthesizes findings from 53 preclinical and 18 clinical studies published between 2015 and 2025, providing a first-of-its-kind analysis that integrates bioink engineering principles with NSC-specific regenerative cues in neurosurgical applications. Our findings highlight that scaffold vascularization, immune compatibility,and GMP-standardized manufacturing remain the most pressing translational challenges,yet convergence of these technologies has the potential to redefine functional CNS repair. Despite promising outcomes, clinical translation faces challenges including cell survival, vascular integration, scaffold standardization,and regulatory compliance. Emerging technologies such as 4D bioprinting,CRISPR-engineered NSCs,and organoid-on-a-chip platforms are poised to overcome these barriers. By integrating structural bioengineering with cellular therapy, this interdisciplinary approach holds transformative potential for CNS repair. This review aims to provide a comprehensive overview of current advancements, translational bottlenecks,and future directions in regenerative neurosurgery using bioprinted NSC-based constructs.

## 1 Introduction

Neurosurgery is a highly specialized medical discipline focused on the diagnosis, surgical management, and rehabilitation of disorders affecting the central nervous system (CNS), including the brain and spinal cord, as well as the peripheral nervous system (Sofroniew and Vinters, 2010). Advances in microsurgical techniques, neuronavigation, neuroimaging, and intraoperative monitoring have significantly improved surgical precision and perioperative outcomes over the past decades (Bradbury and Burnside, 2019). Despite these technological developments, neurosurgical interventions remain fundamentally limited by the poor intrinsic regenerative capacity of the CNS (Carmichael, 2016). Following trauma, ischemic or hemorrhagic stroke, neurodegenerative disease, or tumor resection, neural tissue exhibits minimal spontaneous repair, resulting in permanent functional deficits (Raineteau and Schwab, 2001). In this context, 3D bioprinting has emerged as a bioengineering strategy to address the unmet requirements of stem cell–based neural regeneration, rather than as a surgical tool. As an advanced additive manufacturing technology, 3D bioprinting enables the fabrication of customized, cell-laden constructs through the precise spatial deposition of biocompatible bioinks (Kerschensteiner et al., 2005; Gage and Temple, 2013). Unlike conventional injection-based delivery or simple hydrogels, bioprinted scaffolds allow controlled cell localization, mechanical tuning, biochemical patterning, and multicellular organization—features that are critical for recreating biomimetic neural niches (Ming and Song, 2011; Lindvall and Kokaia, 2010). These engineered constructs can support NSC viability, direct lineage commitment, promote vascularization, and facilitate host–graft integration in ways that are not achievable with existing delivery methods.

To address this biological limitation, regenerative medicine approaches particularly stem cell–based therapies have emerged as promising adjuncts to conventional neurosurgical care, rather than replacements for surgery itself. Neural stem cells (NSCs) possess the capacity for self-renewal and multipotent differentiation into neurons, astrocytes, and oligodendrocytes, enabling cell replacement, trophic support, immunomodulation, and angiogenesis (Barker et al., 2018; Martino and Pluchino, 2006; Goldman and Kuypers, 2015). Preclinical studies and early-phase clinical trials have demonstrated the therapeutic potential of NSCs in conditions such as spinal cord injury, Parkinson’s disease, multiple sclerosis, stroke, and traumatic brain injury (Pluchino and Cossetti, 2015). Importantly, these regenerative strategies aim to restore neural function after surgical or pathological damage, rather than to assist surgeons during operative procedures. The global incidence of CNS injuries and degenerative disorders underscores this urgency: SCI affect an estimated 250,000–500,000 people annually, Parkinson’s disease affects over 10 million worldwide, and traumatic brain injury impacts nearly 69 million individuals each year. These conditions impose a combined global economic burden exceeding USD 500 billion annually through direct healthcare costs, loss of productivity, and long-term rehabilitation.

However, despite their biological promise, standalone NSC therapies have produced inconsistent and often modest clinical outcomes. Major limitations include poor survival of transplanted cells in hostile post-injury environments, uncontrolled migration, inadequate differentiation, limited synaptic integration, and immune-mediated rejection (Tuszynski and Steward, 2012). These challenges highlight that the primary bottleneck in NSC therapy is not the therapeutic cells themselves, but the absence of a controllable and supportive microenvironment that can guide their survival, organization, and functional integration within damaged CNS tissue. Thus, 3D bioprinting should be viewed not as a requirement for neurosurgical procedures, but as a complementary regenerative platform designed to overcome the biological and translational limitations of neural stem cell therapy. The convergence of NSC biology and 3D bioprinting represents an interdisciplinary effort to enhance post-surgical neural repair and functional recovery. This review critically examines the current status of NSC therapy, the challenges limiting its clinical translation, and the rationale for integrating 3D bioprinting as a supportive strategy in regenerative neurosurgery. Whereas 3D bioprinting creates the structure part of tissue engineering, neural stem cell (NSC) therapy adds the needed biological dynamics of functional regeneration (Barker et al., 2018). NSCs themselves possess the intrinsic ability to self-renewal and multipotent differentiation, and potentially form neurons, astrocytes, and oligodendrocytes (Martino and Pluchino, 2006). These cells are important in restoring neural networks, regulation of the inflammatory responses, stimulating angiogenesis, and restoration of lost or harmed cells (Goldman and Kuypers, 2015). The preclinical studies and early clinical trials have revealed the possibility of living cells of NSCs in treating the spinal cord injuries (SCI) and Parkinson diseases, multiple sclerosis and other CNS disorders (Pluchino and Cossetti, 2015). One of the greatest shortcomings of standalone stem cell therapy, however, is the fact that the localization of the cells, the differentiation stimuli, and integration of the host tissue cannot be controlled (Tuszynski and Steward, 2012). While both 3D bioprinting and NSC therapy have individually shown promise, no comprehensive strategy has yet emerged that fully integrates these approaches for targeted neurosurgical applications a gap this review aims to address (Lindvall and Kokaia, 2010; Barker et al., 2018; Martino and Pluchino, 2006; Goldman and Kuypers, 2015; Pluchino and Cossetti, 2015).

**FIGURE 1:**
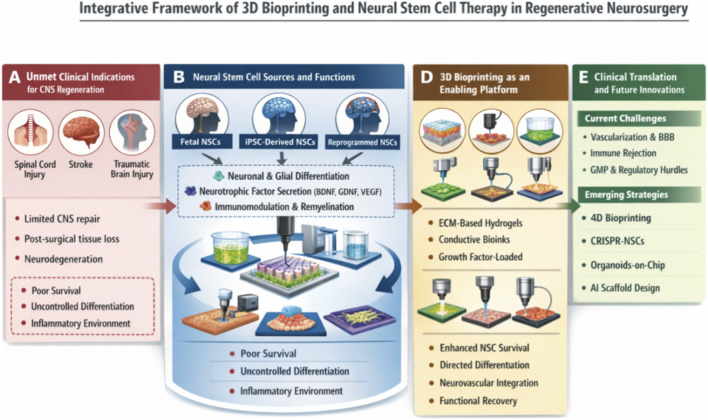
Integrative strategy of 3D bioprinting and NSC therapy in neurosurgery.

In order to address this, intra-scaffold integration of NSCs in 3D bioprinted scaffolds emerges to be a synergistic approach one that embodies the mechanical and architectural precision of bioprinting and the regenerative potential of stem cells (Trounson and McDonald, 2015). The scaffold in such systems hosts a beneficial biomimetic niche to NSCs favoring their viability, their differentiation, and spatially orienting the reconstruction of neural networks. This interventional strategy has bridged new frontiers in regenerative neurosurgery and the limits of what can be done in restoration and functional restoration of neural tissues (Mandrycky et al., 2016). With these trends in mind, this current review will critically discuss how 3D bioprinting and NSC have been converging to be used in neurosurgical treatment (Malilay et al., 2020). As an illustrative representation of the interdisciplinary framework dealt with, Figure 1 depicts a conceptual representation of the interconnection between 3D printing of bioprinted cells and the treatment of NSC therapy and how the synergy is being converged to address all the relevant neurosurgical challenges and achieve therapeutic deliberate regeneration. We consider the basic science supporting each technology, give an overview of recent results of preclinical and clinical outcomes, and comment on the issues of translation and opportunity (Gillett et al., 2021). Scaffold design, biocompatibility, cell-scaffold interaction, immune responses as well as strategies of vascularization are noted specifically. Finally, the goal of this review is to offer an in-depth knowledge of this new paradigm and define future research avenues that can potentially become clinically feasible in finding solutions to CNS repair using integrative regenerative technologies (Geiger and Hirschl, 2015).

### 1.1 Search strategy

A comprehensive literature and clinical trial search was conducted to identify relevant studies focusing on neural stem cell (NSC) based therapies, biomaterial scaffolds, and 3D bioprinting applications in neural tissue engineering and neurosurgical repair. The search was carried out in accordance with PRISMA guidelines across multiple databases, including PubMed/MEDLINE, Scopus, Web of Science, and Embase, as well as clinical trial registries such as ClinicalTrials.gov and the WHO International Clinical Trials Registry Platform (ICTRP). The search period spanned from January 2015 to September 2025. Both controlled vocabulary (MeSH terms) and free-text keywords were used in combination with Boolean operators. A representative PubMed search string included: (“neural stem cells” OR “neuroprogenitor cells” OR “mesenchymal stem cells” OR “iPSC” OR “embryonic stem cells”) AND (“bioprinting” OR “scaffold” OR “hydrogel” OR “neural tissue engineering”) AND (“neurosurgery” OR “spinal cord injury” OR “stroke” OR “Parkinson’s disease” OR “traumatic brain injury” OR “clinical trial”). Reference lists of key publications and relevant reviews were also screened to identify additional studies.

#### 1.1.1 Inclusion and exclusion criteria

Encompassed original research articles, preclinical and clinical trials, and systematic reviews focusing on NSC or scaffold-based neural repair published in English. Studies were required to involve human participants or mammalian preclinical models and report outcomes related to neural regeneration, functional recovery, graft integration, or translational feasibility. Exclusion criteria included non-neural tissue applications, cosmetic bioprinting, editorials, conference abstracts lacking full data, and non-peer-reviewed sources. The selection process involved two stages: initial screening of titles and abstracts for relevance, followed by full-text review for eligibility confirmation. Discrepancies in study inclusion were resolved through consensus among the authors.

#### 1.1.2 Results

Overall, the search initially identified 184 studies and 22 registered clinical trials. A total of 98 publications were screened, of which key preclinical and clinical studies (including 12 clinical trials) were qualitatively discussed in this review. The process of identification, screening, and inclusion was summarized in a PRISMA flow diagram (Figure 1).

## 2 Engineering requirements imposed by neural stem cell therapy: why 3D bioprinting is needed

Three-dimensional (3D) bioprinting has emerged as a transformative bioengineering approach in neural tissue engineering, enabling the fabrication of spatially controlled, biomimetic constructs that recapitulate key architectural and biochemical features of the central nervous system (CNS) (Pati et al., 2016). This technology integrates biofabrication techniques with biologically active materials and viable cells to generate engineered neural grafts designed to support cell survival, differentiation, and network formation. Although 3D bioprinting is not yet implemented as a routine tool in clinical neurosurgical practice, it has demonstrated substantial potential in preclinical and translational studies to mechanically support injured tissue, guide axonal regeneration, and enhance cellular integration following traumatic injury, neurodegenerative disease, or surgical resection (Zhang et al., 2017). As such, 3D bioprinting is currently positioned as an enabling regenerative platform rather than a direct operative technology in neurosurgery.

### 2.1 Bioinks and scaffold design

Bioink and scaffold composition designs are vital in promoting biocompatibility, mechanical stability, and functionality fusion of the printed neural constructs. The ability of different types of hydrogel-based bioinks to trap NSCs and nurture neural differentiation has been tested (Kosterhon et al., 2020). Composites of alginate, agarose and carboxymethyl chitosan have been introduced as potential bioinks as they exhibit desirable rheological properties as well as cell-compatibility. These bioinks proved to have organized distribution of printed cells, high viability after printing and characterization of supporting neural differentiation with functional characteristics such as calcium signalling, which implies living neuronal networks (Hinton et al., 2015) when printed with NSCs. Extracellular Matrix (ECM)-synthesized hydrogels, especially the ones that are made of decellularized nerve tissue and crosslinked with genipin, provide biomimetic microenvironment that stimulates neurite growth and enables increased myelination. Such hydrogels lend themselves especially well to extrusion-based bio-printer applications and have been successful in preclinical trials of patterns related to the repair of peripheral and CNSs. Besides this, conductive hydrogel-based bioinks, e.g., graphene nanoplatelet- or polyaniline-blending bioinks are also being developed to replicate the electrochemistry of the nervous tissue (Zhang et al., 2019). Such scaffolds have an electrical conductivity and brain like mechanical properties making them fundamental in the promotion of axonal regeneration, boosting of synaptic activity, and remodeling functional connectivity in injured neural networks. According to the discussion put forward in Table 1, all the scaffolds.

**TABLE 1:** Comparative properties of common bioinks in neural tissue bioprinting.

S.No.	Bioink type	Biocompatibility	Electrical conductivity	Support for NSC differentiation	Printability	Key applications	References
1	Alginate–Agarose–Chitosan	High	Low	Moderate	High	Basic neural scaffolds	Hinton et al. (2015)
2	ECM-derived (Decellularized)	High	Low	High	Moderate	Myelination and neurite guidance	Pati et al. (2014)
3	GelMA + Graphene	High	High	High	Moderate	Functional synapse formation	Jakus et al. (2015)
4	Fibrin-based with VEGF	High	Low	High (with cues)	Low	Angiogenesis, neurovascular interfaces	Li et al. (2020)
5	Conductive polymers (e.g., PANi)	Moderate	High	High	Moderate	Electrical stimulation, axon bridging	Balint et al. (2014)

### 2.2 Fabrication techniques and functional architectures

Among the different bioprinting modalities, extrusion-based bioprinting (EBB) is currently the most widely adopted technique for neural tissue engineering. It allows for the deposition of a wide range of viscous bioinks and supports high cell densities (Zhang et al., 2017). However, EBB faces challenges in achieving microscale resolution (<50 μm) and maintaining architectural fidelity, especially when replicating the intricate microanatomy of neural tissues (Panda et al., 2022). A notable advancement in this field is the development of a horizontal bioprinting platform, which enables the stratified printing of human cortical and striatal progenitor cells in a layer-specific manner. This approach has led to the successful formation of 3D constructs containing regionally distinct neural populations (Zhang et al., 2019). Recent developments, such as horizontal and layer-specific bioprinting, have advanced the creation of constructs mimicking cortical and striatal organization (Panda et al., 2022). These 3D constructs have demonstrated spontaneous neuronal maturation, electrophysiological activity, and glial–neuronal interactions, closely resembling native brain microenvironments. Within weeks, these constructs demonstrated spontaneous maturation, functional neuronal activity, and synaptic interactions between neurons and glial cells, recapitulating key features of native brain circuits (Trucco et al., 2021). These advances underscore the potential of 3D bioprinting not only for tissue replacement but also for modelling neurodevelopmental processes and disease pathophysiology *in vitro* (Trucco et al., 2021). The integration of computer-aided design, patient-specific cell sourcing, and functional bioinks has paved the way for the development of personalized neural tissue constructs (Züger et al., 2023). From a neurosurgical perspective, these innovations represent a step toward patient-specific neuroregenerative therapy. Personalized constructs could aid in repairing cortical lesions, spinal cord injuries, and stroke-induced neural loss (Züger et al., 2023). From a neurosurgical perspective, these innovations represent a step toward patient-specific neuroregenerative therapy by enabling the design of anatomically matched, lesion-conforming constructs tailored to individual injury patterns. In practical terms, patient imaging data (magnetic resonance imaging or computed tomography) can be used to generate three-dimensional defect maps, which guide the fabrication of bioprinted scaffolds with defined geometry, stiffness, and cellular composition. Following standard neurosurgical exposure and lesion debridement, such constructs are intended for direct implantation into cortical cavities, stroke-induced infarct zones, or spinal cord lesion gaps, where they serve as a physical bridge to support axonal growth and host–graft integration rather than replacing operative repair itself (Züger et al., 2023).

In addition to *in vivo* implantation strategies, 3D bioprinted neural tissues are increasingly used as *ex vivo* and *in vitro* platforms for neurosurgical applications, including modeling patient-specific lesion architecture, simulating tissue–implant interactions, and testing neuroprotective or neuroregenerative agents prior to clinical translation. These bioprinted models allow controlled evaluation of cellular survival, inflammatory responses, and electrophysiological integration under conditions that approximate the post-surgical CNS microenvironment. Collectively, these approaches position 3D bioprinting as a supportive regenerative adjunct to neurosurgical intervention, rather than a replacement for established operative techniques.

**FIGURE 2:**
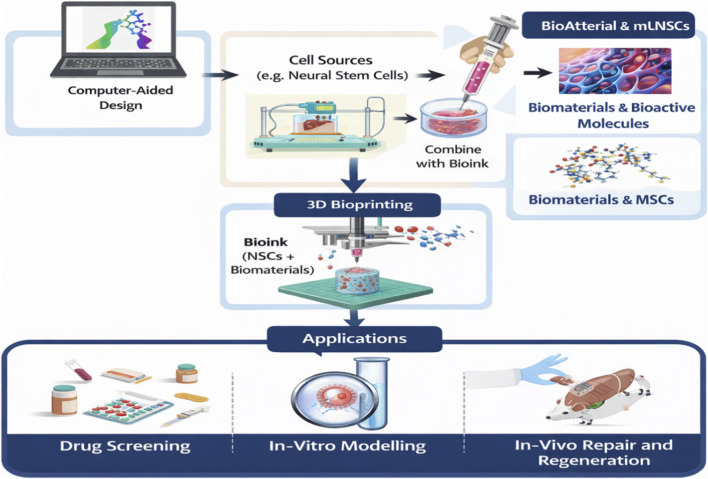
Workflow of 3D bioprinting in neural tissue engineering and regenerative medicine.

By integrating computer-aided design (CAD), stem-cell-derived bioinks, and precision bioprinting, these technologies move closer to clinical translation, where construct integration, vascularization, and functional restoration remain key challenges (Rolston et al., 2014). Figure 2 presents a conceptual workflow illustrating how 3D bioprinting is applied in neural tissue engineering, from component preparation to applications in drug testing, disease modeling, and CNS repair. A concise comparison of various fabrication modalities, their advantages, limitations, and neurosurgical relevance is summarized in Table 2.

**TABLE 2:** Summary of 3D bioprinting techniques and their neurosurgical relevance.

S.No.	Bioprinting technique	Technical principle	Typical bioinks/Cell types	Neurosurgical application (experimental/Clinical)	Feasibility level	Key references
1	Extrusion-Based Bioprinting (EBB)	Continuous extrusion of viscous bioinks through pneumatic or mechanical pressure	GelMA, alginate, fibrin, dECM hydrogels with NSCs or iPSC-derived neural progenitors	lesion-bridging scaffolds implanted after laminectomy in rodent models; Stroke: cavity-filling constructs post-infarct; TBI: cortical defect filling after craniotomy	High (preclinical), early translational	Trucco et al. (2021), Züger et al. (2023)
2	Inkjet Bioprinting	Droplet-based deposition of low-viscosity bioinks with high spatial precision	Low-density hydrogels, growth-factor-loaded bioinks, neural progenitors	Experimental neurosurgery: micro-patterning of neuronal networks for *in vitro* cortical models; Drug testing: synaptic toxicity and neuroprotection screening	Moderate (*in vitro*/*ex vivo*)	Li et al. (2020), Züger et al. (2023)
3	Laser-Assisted Bioprinting (LAB)	Laser-induced forward transfer of cell-laden droplets without nozzle contact	High-density NSCs, neural progenitors, endothelial cells	Experimental CNS repair: precise placement of NSCs at lesion margins in SCI and stroke models; Cortical mapping: high-resolution neuronal patterning	Moderate (advanced preclinical)	Trucco et al. (2021), Züger et al. (2023)
4	Stereolithography (SLA/DLP)	Photopolymerization of light-sensitive bioinks with high resolution	PEGDA, GelMA with neural progenitors	Neurosurgical modeling: fabrication of transparent, anatomically accurate brain tissue constructs for disease modeling and surgical simulation	Low–Moderate (*in vitro*)	Trucco et al. (2021), Züger et al. (2023)
5	Hybrid Bioprinting (Multimodal)	Combination of extrusion + inkjet or SLA for multi-material constructs	NSCs + endothelial cells + conductive bioinks	SCI and stroke: vascularized neural grafts implanted into lesion cavities; Clinical feasibility studies: scaffold-assisted NSC delivery	Emerging translational	Züger et al. (2023)

Different 3D bioprinting techniques offer distinct advantages and limitations that determine their feasibility for neurosurgical applications. Among these, extrusion-based bioprinting currently represents the most practical approach for neural tissue engineering due to its compatibility with high-viscosity, cell-laden hydrogels and its ability to fabricate centimeter-scale constructs suitable for implantation. In experimental neurosurgery, extrusion-bioprinted NSC-loaded scaffolds have been implanted directly into spinal cord lesion gaps following laminectomy, where they function as physical bridges to support axonal regeneration and host–graft integration (Panda et al., 2022; Trucco et al., 2021; Züger et al., 2023). Similar strategies have been explored in stroke and traumatic brain injury models, in which bioprinted constructs are shaped to fill post-infarct or post-resection cavities after standard neurosurgical exposure.

Inkjet and laser-assisted bioprinting techniques, while less suited for bulk tissue replacement, provide superior spatial resolution and are primarily applied in experimental and translational settings, such as micro-patterning neural circuits, studying synaptic organization, and evaluating neuroprotective drugs. Stereolithography-based approaches are mainly used to generate high-resolution, optically transparent neural constructs for *in vitro* disease modeling and neurosurgical simulation rather than direct implantation (Panda et al., 2022; Trucco et al., 2021; Züger et al., 2023; Rolston et al., 2014). Hybrid bioprinting strategies that integrate multiple printing modalities are emerging as promising platforms for producing vascularized and multicellular neural grafts, addressing key translational barriers such as nutrient diffusion and graft survival. Collectively, these techniques position 3D bioprinting as a supportive regenerative adjunct to neurosurgical intervention, with current feasibility strongest in experimental models and early translational studies rather than routine clinical practice.

### 2.3 Limitations and future directions

Despite significant progress, several technical and biological limitations hinder the clinical translation of bioprinted neural constructs. One of the most pressing challenges is the lack of integrated vascular networks within printed tissues (Rolston et al., 2014). Without sufficient vascularization, large constructs are prone to central necrosis due to insufficient nutrient and oxygen diffusion. Integrating perfusable synthetic vasculature, angiogenic cues, and even components of the blood–brain barrier (BBB) into neural scaffolds remains a key area of ongoing research. Furthermore, there is a critical need for standardized characterization protocols to evaluate the mechanical strength, electrical conductivity, metabolic activity, and long-term biocompatibility of neural bioprinted constructs (Harput et al., 2014). Without such benchmarks, comparing outcomes across studies or validating constructs for drug screening or clinical use becomes difficult (McGuire et al., 2021). Looking ahead, future strategies will likely involve multi-material bioprinting, real-time bioprinting in surgical settings, and bio-fabrication of innervated, vascularized, and immunologically compatible neural tissues. Continued advancements in bio ink chemistry, printing resolution, and scaffold functionalization are essential to bridge the gap between experimental success and clinical application (Haemmerli et al., 2021). Due to heterogeneity in study design, outcome measures, and reporting standards, a quantitative meta-analysis or statistically weighted aggregation was not feasible. Figures summarizing trends are therefore descriptive and intended to illustrate overarching patterns rather than precise distributions. Wherever possible, original experimental and clinical studies were prioritized over secondary review articles, particularly in tabulated summaries. Review articles were cited selectively to provide conceptual context.

### 2.4 Emerging innovations in bioink development for neural applications

Current dynamic and multifunctional bioinks are new hallmarks of material evolution with dynamics in their structure, both in their roles as supple enabling materials and in their material applications, resulting in bioinks with adaptive features. Intelligent bioinks that include the use of thermo-sensitive polymers such as poly(N-isopropylacrylamide) (PNIPAM) make it possible to gel *in situ* after implantation, increasing scaffold integration and filling irregular CNS defects well (Lee et al., 2012). Also, shear-thinning nanocomposite bioinks that includes carbon nanotubes or MXene nanosheets offer mechanical support and electrical conductivity that can be adjusted to resemble more closely that of the native brain tissue. RGD or IKVAV sequences as an example of bioactive peptide has been found to be directly incorporated into the hydrogel network to increase NSC adhesion, neurite growth and synaptic connections. Further, bioinks loaded with oxygen-entrapping microspheres or angiogenic NPs (e.g., VEGF-loaded PLGA) can be used to pro-actively combat hypoxic stress in large constructs. Table 3 will provide an overview of most recent generation of neural bioinks, their composition, functionality, and translational maturity (Curtis et al., 2018; Tuszynski et al., 2014).

**TABLE 3:** Recent advances in neural tissue-specific bioinks.

S.No.	Bioink composition	Special feature	Electrical conductivity	NSC support	Translational status
1	PNIPAM-GelMA blend	Thermo-responsive gelation	Low	High	Preclinical
2	MXene-GelMA	High conductivity, antioxidant	High	High	Lab-scale
3	RGD-functionalized Alginate	Enhanced cell adhesion	Low	High	Preclinical
4	Oxygen-releasing hydrogel	Reduces hypoxia	Low	Moderate	Prototype
5	VEGF-PLGA hydrogel	Promotes angiogenesis	Low	High	Preclinical

## 3 Neural stem cell therapy and 3D bioprinting in neurosurgery

NSC therapy has rapidly evolved into a cornerstone of regenerative strategies for CNS repair. The intrinsic ability of NSCs to differentiate into neurons, astrocytes, and oligodendrocytes, coupled with their neurotrophic, immunomodulatory, and angiogenic properties, positions them as promising candidates for restoring function in various neurological conditions (Lee et al., 2012). Parallelly, 3D bioprinting has emerged as a complementary technology that allows for spatially controlled, structurally supportive, and functionally biomimetic constructs to guide tissue regeneration. Together, the integration of NSC therapy and 3D bioprinting marks a significant shift from symptomatic management to tissue-level repair and functional recovery in neurosurgical applications (Curtis et al., 2018). From SCI and traumatic brain injury (TBI) to neurodegenerative diseases like Parkinson’s disease (PD) and amyotrophic lateral sclerosis (ALS), recent preclinical and early clinical studies (2015–2025) have demonstrated the feasibility, safety, and regenerative potential of combining NSCs with bioengineered platforms (Tuszynski et al., 2014). This section explores the converging mechanisms, innovations, and translational pathways that underpin this evolving field (Kalladka et al., 2016).

### 3.1 Preclinical and early clinical advances (2015–2025)

In recent years, translational progress has shifted from observational paradigms to mechanistically informed interventions. For example, researchers demonstrated that human NSCs transplanted into a rodent model of SCI promoted significant locomotor recovery through remyelination and synaptic integration across the lesion site (Glass et al., 2012). Building on such work, a 2020 Phase I clinical trial by *in-vivo* Therapeutics deployed NSC-loaded scaffolds in thoracic SCI patients, reporting procedural safety and mild sensory gains over 12 months. In models of TBI, transplantation of iPSC-derived NSCs enhanced neuroprotection and cognitive recovery through modulation of microglial phenotypes and BDNF secretion (Martino et al., 2011). More recently, embedded NSCs in conductive hydrogels to stimulate electrophysiological regeneration across SCI lesions, significantly improving motor recovery in rats (Martino et al., 2011). In the clinical context, researchers led a pioneering human study transplanting iPSC-derived dopaminergic progenitors into PD patients, observing safe integration and preliminary motor improvements over a 4-year follow-up (Barker et al., 2017). Similarly, a Phase II ALS trial (2022–2024) using intrathecal mesenchymal stem cells demonstrated a deceleration in disease progression and respiratory decline, albeit with variable inter-patient outcomes (Kirkeby et al., 2018). A summary of the clinical trials is presented in Table 4.

**TABLE 4:** Summary of key preclinical and clinical studies using NSCs (2015–2025).

S.No.	Model/Population	NSC source	Delivery method	Key functional outcomes	Study type	Reference
1	Mouse thoracic SCI	iPSC-derived NSCs	Intraspinal injection	Significant locomotor recovery, remyelination, synapse formation	Preclinical	Nori et al. (2011)
2	Rat SCI	Human fetal NSCs	Intraspinal injection	Axonal regeneration, improved BBB locomotor scores	Preclinical	de Freria et al. (2021)
3	Chronic ischemic stroke/SCI overlap	Human NSCs	Intracerebral/intraspinal	Functional improvement, safety confirmed	Clinical (Phase I)	Curtis et al. (2018)
4	Chronic cervical SCI (humans)	Human spinal cord–derived NSCs	Direct intramedullary injection	Sensory improvement, stable graft survival	Clinical (Phase I)	Lee et al. (2022)

Neural stem cell transplantation has demonstrated the capacity to promote functional recovery following spinal cord injury, primarily through mechanisms involving remyelination, synaptic integration, and modulation of the post-injury inflammatory microenvironment. Preclinical studies consistently report improved locomotor outcomes, while early-phase clinical trials have established procedural safety and modest sensory or motor gains, underscoring both the therapeutic promise and current translational limitations of NSC-based interventions (Nori et al., 2011; de Freria et al., 2021; Curtis et al., 2018; Lee et al., 2022).

### 3.2 Mechanistic insights and translational innovations

Neural stem cell (NSC) therapy contributes to central nervous system repair through multiple complementary mechanisms, not only cell replacement. In preclinical models, NSCs can support neurorepair by releasing neurotrophic and angiogenic mediators (for example, brain-derived neurotrophic factor, glial cell line-derived neurotrophic factor, and vascular endothelial growth factor), and by shaping the inflammatory milieu toward a more permissive, pro-repair state. To improve cell survival and integration, increasing attention has shifted from “cells alone” to cells delivered within engineered niches (Jiu et al., 2024). In this approach, biomaterial scaffolds (including 3D-bioprinted constructs) provide (1) mechanical stabilization, (2) controlled cell localization, and (3) biochemical and electrical cues that can guide neurite extension and network formation—features that are difficult to achieve with injection-only delivery. Recent spinal cord injury studies using 3D-bioprinted, tissue-mimetic scaffolds report enhanced structural bridging and functional recovery compared with non-printed or non-conductive controls (Jiu et al., 2024). Finally, immuno-engineering strategies are being explored to further reduce secondary injury cascades. While many “IL-10 overexpression” examples in traumatic brain injury are mesenchymal stromal cell (MSC) studies rather than NSCs, these data still illustrate how local delivery of anti-inflammatory signals can reduce gliosis and improve functional outcomes—an approach that can be adapted to NSC-based platforms when safety and differentiation control are ensured (Liu et al., 2021) (Table 5).

**TABLE 5:** Recent studies combining NSCs with 3D bioprinting for CNS repair (2023–2025).

S.No.	Bioprinted construct/Scaffold	NSC source/Model	Injury/Model	Key outcomes	Reference
1	SC-dECM hydrogel + NSCs + TGF-β1 antibody	NSCs (rat)	Spinal cord injury (rat complete transection)	Improved motor function (BBB score), enhanced neuronal differentiation, reduced glial scarring	Deng et al. (2024)
2	Aligned GG-LCS-NSC 3D bioprinted constructs	NSC-laden constructs	Complete transection SCI (rat)	Promoted locomotor recovery, increased neuronal regeneration, enhanced angiogenesis	Zhang et al. (2024)
3	(Review) Emerging strategies for 3D bioprinted neural tissues	NSCs with patterned constructs	*In vitro* and translational focus	Summarizes spatial organization, host-integration challenges, and future translational directions	Kim et al. (2024)

Recent preclinical advances demonstrate that 3D-bioprinted, NSC-laden scaffolds can improve structural and functional outcomes in central nervous system repair. For example, 3% decellularized extracellular matrix (dECM) hydrogel scaffolds loaded with NSCs and TGF-β1 antibody significantly enhanced motor function and neuronal regeneration in a complete transection spinal cord injury model, indicating improved locomotor recovery and reduced inhibitory scarring (Deng et al., 2024; Zhang et al., 2024; Kim et al., 2024; Mobbs et al., 2017; Tsuji et al., 2010; Mota et al., 2020; Nguyen et al., 2016). Similarly, aligned 3D-bioprinted constructs containing NSCs promoted both axonal regeneration and angiogenesis in rat spinal cord injury models, with superior hindlimb functional scores compared with control constructs. While translational challenges remain, recent systematic analyses of neural bioprinted models underscore progress toward clinically relevant architectures that support cell viability, vascularization, and functional integration (Nguyen et al., 2016; Samadian et al., 2020).

**FIGURE 3:**
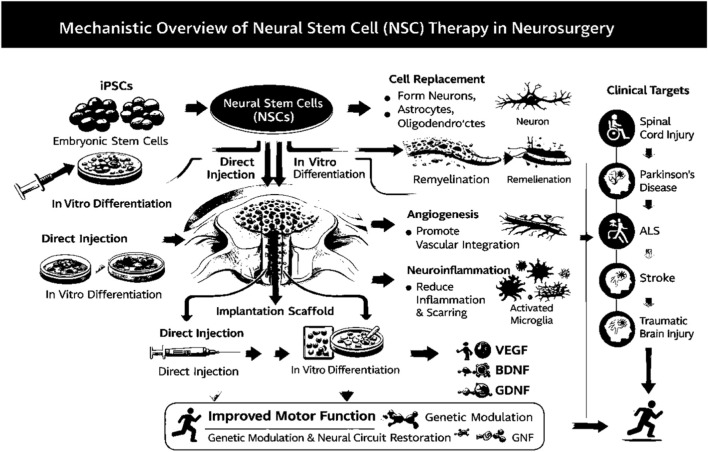
Mechanistic overview of neural stem cell (NSC) therapy in neurosurgery.

Figure 3 provides an overview of NSC-based therapeutic strategies, highlighting the sources, delivery methods, mechanisms, and clinical targets. Stem cells such as iPSCs, embryonic, and mesenchymal cells are used either through direct injection or after *in vitro* differentiation. These cells contribute to neural repair by promoting remyelination, angiogenesis, and reducing neuroinflammation ultimately improving motor function. The therapeutic approach targets conditions like spinal cord injury, Parkinson’s disease, ALS, and TBI, aiming for genetic modulation and neural circuit restoration.

### 3.3 Scaffold parameters shaping NSC fate

Biophysical and biochemical properties of scaffolds play a key role in regulating NSC behavior. Mechanical stiffness influences lineage commitment, where softer matrices such as PEGDA and GelMA tend to support neuronal differentiation, while stiffer environments may promote glial fate (Zhang et al., 2014). Electrical conductivity contributes to axonal conduction and synaptic activity, with materials like graphene and polypyrrole showing promising results in enhancing neural signal transmission. Scaffold compositions that mimic the extracellular matrix, such as decellularized ECM or collagen–laminin blends, help improve NSC viability and support processes like myelination (Li et al., 2021). The incorporation of trophic factors such as VEGF, BDNF, and SDF-1 within the scaffold can create gradients that guide cell migration and support vascularization (Palomeque Chávez et al., 2025). Additionally, the alignment of scaffold fibers, achieved through techniques like electrospinning, can direct axonal growth along defined paths, which is crucial for organized neural network formation. This scaffold collectively supports the therapeutic potential of NSCs in neural tissue engineering (Zhao et al., 2021).

### 3.4 3D bioprinted NSC models for disease modeling and drug discovery

Beyond regenerative medicine, bioprinted NSC constructs serve as disease-specific models with high translational value. These platforms enable the study of neural circuit formation, neuroinflammation, and synaptic dynamics under pathological conditions (Yao et al., 2020). For instance, developed a 3D bioprinted cortical model for epilepsy using iPSC-derived cortical progenitors (Ma et al., 2018). The model exhibited spontaneous calcium oscillations and allowed high-throughput antiepileptic drug screening (Ma et al., 2018) (Table 6). Similarly, one of the researchers fabricated an Alzheimer’s disease model using Aβ-laden ECM bioinks, which mimicked amyloid aggregation and synaptic toxicity (Benwood et al., 2023).

**TABLE 6:** Bioprinted NSC constructs for neurological disease research.

S.No.	Disease model	Scaffold composition	NSC type	Application	Reference
1	Epilepsy	PEGDA + cortical progenitors	iPSC-derived NSCs	Seizure modeling, drug testing	Cheng et al. (2012)
2	Alzheimer’s disease	ECM + Aβ peptides	iPSC-derived NSCs	Anti-amyloid drug screening	Trujillo et al. (2018)
3	Glioblastoma invasion	Gelatin–collagen hydrogel	Co-cultured NSCs	Tumor–stroma interaction studies	Marei et al. (2023)
4	SCI	Conductive hydrogel	NSCs + astrocytes	Electrophysiological recovery models	Choi et al. (2014)

Immune compatibility remains a critical factor for the long-term survival and functional success of transplanted neural stem cells (NSCs). Even when autologous or human leukocyte antigen (HLA)–matched induced pluripotent stem cell (iPSC)–derived NSCs are used, host immune responses can still occur. Activation of resident microglia and astrocytes often leads to neuroinflammation, gliosis, and scar formation, which together create a physical and biochemical barrier that limits axonal regeneration and graft integration (Cheng et al., 2012; Trujillo et al., 2018).

To address these challenges, recent research has focused on modifying biomaterial scaffolds to locally regulate immune responses. Incorporation of immunomodulatory molecules, such as anti-inflammatory cytokines (e.g., interleukin-10 or transforming growth factor-β), into scaffold matrices has been shown to suppress the local release of pro-inflammatory mediators while preserving tissue repair processes (Pati et al., 2014; Kerschensteiner et al., 2005). Additionally, the inclusion of heparin within scaffolds can enhance the retention and sustained release of anti-inflammatory and pro-regenerative factors, thereby improving the local microenvironment for NSC survival (Yao et al., 2020).

Beyond biochemical strategies, physical properties of scaffolds also play an important role in immune modulation. Nanoscale surface features, such as aligned grooves in the range of 50–100 nm, have been reported to reduce pro-inflammatory (M1) macrophage activation while promoting anti-inflammatory (M2) phenotypes that support neural repair and regeneration (Balint et al.; Bakhtiiari et al., 2021; Nguyen et al., 2016). Collectively, these immunomodulatory scaffold strategies represent an important approach to improving graft survival, reducing inflammation, and enhancing functional outcomes in neural tissue engineering.

Table 7 has highlighted immunomodulatory strategies of use in NSC-bioprinted constructs.

**TABLE 7:** Immunomodulatory strategies for neural tissue bioprinting.

S.No.	Strategy	Mechanism	Outcome	Model/Status
1	IL-10 tethered hydrogel	Local cytokine suppression	Reduced gliosis	Rat SCI model
2	Heparinized ECM scaffold	Growth factor retention	Enhanced angiogenesis	Preclinical
3	Nano-grooved PCL fibers	Macrophage M2 bias	Reduced inflammation	*In vitro*
4	MSC co-seeding	Immunomodulatory secretome	Increased NSC survival	Mouse TBI
5	Decellularized ECM + FK506	T-cell inhibition	Improved graft integration	​

## 4 Clinical translation: opportunities and hurdles

While NSC therapy and 3D bioprinting have demonstrated significant promise in preclinical studies, clinical translation presents nuanced challenges that span safety, scalability, regulatory compliance, and therapeutic consistency (Liang, 2023). As of 2025, over 20 early-phase clinical trials have explored NSC-based interventions for CNS injuries and neurodegenerative diseases, yet the leap to widespread clinical adoption remains constrained by biological variability and translational inefficiencies (Bliley et al., 2022).

### 4.1 Clinical maturity of NSC-Based interventions

Clinical trials for SCI and PD have yielded encouraging yet modest results. For instance, a Phase I trial using iPSC-derived dopaminergic progenitors in PD patients revealed safe integration with mild motor improvement. However, long-term functional gains remain inconsistent across cohorts (Sawamoto et al., 2025; Tabar et al., 2025; Bruschettini et al., 2023; Schweitzer et al., 2020). For instance, a Phase I trial using induced pluripotent stem cell (iPSC)-derived dopaminergic progenitors in Parkinson’s disease demonstrated safe graft survival and mild motor improvement, but the functional gains were not sustained beyond 24 months, underscoring the challenges of neuronal integration and host–graft communication. Similarly, a recent meta-analysis reported that among 11 SCI trials, only four demonstrated statistically significant motor function recovery, with cell engraftment and host integration being key limiting variables.

However, several biological and operational barriers continue to impede widespread clinical adoption. Variability in differentiation protocols, batch-to-batch inconsistency, and limited scalability of GMP-grade NSC production remain pressing concerns. Additionally, immune compatibility and long-term graft survival have emerged as key determinants of clinical success, with immune rejection observed even in autologous or HLA-matched models (Manjili, 2025). To address these gaps, current translational pipelines are increasingly adopting closed-loop bioreactors, AI-assisted quality control systems, and standardized cryopreservation methods to improve reproducibility and regulatory compliance. Collectively, these developments mark a gradual but significant transition from proof-of-concept to clinically guided optimization of NSC therapies. The integration of real-world clinical data with biomanufacturing innovations represents a crucial step toward scalable, safe, and functionally effective neurorestorative interventions.

### 4.2 Regulatory and manufacturing challenges

The regulatory landscape for NSC and bioprinted therapies is still fragmented and evolving. While the U.S. FDA’s RMAT designation has expedited some therapies, such as BrainStorm’s NurOwn for ALS, most NSC interventions fall short of the stringent Good Manufacturing Practice (GMP) criteria for long-term approval (Schweitzer et al., 2020). A major challenge lies in standardizing bioprinted constructs, including the reproducibility of scaffold architecture, cell viability during printing, and sterility maintenance across batches (Manjili, 2025). Key barriers include batch-to-batch variability, sterility control during scaffold fabrication, and inconsistent differentiation efficiency across production lines. Meanwhile, a 2024 white paper from the European Medicines Agency (EMA) emphasized the need for 3D bioprinting-specific quality assurance standards, especially for constructs involving dynamic (“4D”) elements (Lin et al., 2025). Ethical concerns also linger, particularly around the sourcing of fetal NSCs and genome-edited lines. From a manufacturing standpoint, automation and closed-system bioreactors have begun to address contamination risks and variability by allowing real-time monitoring of oxygenation, nutrient flow, and differentiation status. However, the cost burden of GMP-grade bioinks and scaffolds remains high, with single patient-specific constructs costing up to USD 15,000. Standardized quality-control pipelines, shared biorepositories, and harmonized international regulatory guidelines will be crucial to bridge the gap between lab-scale prototypes and clinically deployable neuro-constructs (Sekar et al., 2021; Mladenovska et al., 2023; Sadiq et al., 2025; Verma, 2023; Chang, 2023; Kelly and Vermeulen, 2017).

### 4.3 Strategic opportunities

Despite hurdles, several translational enablers are emerging:

- Personalized regenerative platforms: The use of *iPSC-derived NSCs* matched to patient *HLA profiles* significantly reduces immune rejection, forming the foundation for personalized graft therapies.
- Smart bioreactors and digital twins: Closed-loop bioreactors integrated with biosensors and AI-driven feedback systems now enable real-time control of cell viability, differentiation, and scaffold maturation—key for clinical reproducibility.
- Bioelectronic and magneto-responsive scaffolds: These enable *non-invasive post-implant modulation* of neuronal activity, potentially improving graft functionalization after transplantation.
- Neuro-surgical integration platforms: Advances in *intraoperative imaging* and *robotic guidance systems* now facilitate precise placement of NSC-laden constructs, reducing procedural variability and improving graft retention.

Together, these innovations represent a clear movement toward translational convergence, where regenerative cell therapy aligns with neurosurgical precision, device engineering, and digital monitoring shifting NSC therapy from experimental biology to clinically actionable neuro-repair.

### 4.4 Future perspectives

Table 8 summarizes key innovations such as 4D bioprinting, organoid-on-a-chip models, CRISPR-engineered NSCs, and hybrid electroceutical-cell systems, highlighting their potential impact on personalized and adaptive neural repair, along with current development status.

**TABLE 8:** Emerging technologies shaping the future of neural stem cell-based therapies.

S.No.	Advancement	Potential impact	Development status	References
1	4D Bioprinting	Stimuli-responsive constructs that evolve *in vivo* for adaptive neural repair	Experimental (MIT & ETH Zurich, 2024)	Sekar et al. (2021)
2	Organoid-on-a-Chip Models	Personalized neurosurgical screening and disease modelling using patient-derived cells	Pilot studies in epilepsy and glioblastoma underway	Mladenovska et al. (2023)
3	CRISPR-Engineered NSCs	Enhanced resilience, differentiation, or anti-inflammatory features	Preclinical trials in Alzheimer’s disease models	Sadiq et al. (2025)
4	Hybrid Systems (Electroceutical-Cell Therapy)	Integration of NSCs with bioelectronic interfaces for real-time modulation	Active research funded by DARPA & NIH	Verma (2023)

Moving forward, translational success will depend on three converging priorities:

1. Engineering vascularized and BBB-compatible scaffolds to improve graft survival;
2. Establishing GMP-compliant manufacturing pipelines for hybrid NSC constructs; and
3. Leveraging AI-driven modeling to optimize scaffold geometry and predict long-term functionality. These approaches, when integrated with neurosurgical workflows and clinical imaging, could finally enable NSC-bioprinting therapies to progress from experimental feasibility to routine clinical application. Future perspectives should move beyond algorithmic improvement to emphasize the pathways for translating AI-integrated precision nursing tools into clinical practice. Large-scale validation trials, interoperability with existing surgical monitoring platforms, and cost-benefit analyses are vital for real-world adoption. Furthermore, ethical deployment frameworks and training modules for perioperative staff must be developed to ensure safe, efficient, and equitable AI integration into surgical workflows.

## 5 Clinical translation and market landscape

Despite remarkable progress in NSC therapy and 3D bioprinting, clinical translation remains complex. This section outlines the current status, major bottlenecks, emerging products, regulatory considerations, and evolving market dynamics (Chang, 2023).

### 5.1 Translational challenges

Translating NSC-based constructs into clinical neurosurgery faces biological and technical barriers. This Table 9 outlines major biological and technical barriers affecting the success of NSC therapies, including issues related to cell survival, graft-host integration, scaffold engineering, and blood-brain barrier (BBB) permeability. However, these outcomes remain preliminary, with modest efficacy and limited durability across studies.

**TABLE 9:** Key challenges hindering the clinical translation of NSC-based therapies.

S.No.	Challenge	Description
1	Cell survival	Implanted NSCs often show limited viability and poor integration in hypoxic or inflamed environments
2	Graft-host integration	Synaptic, vascular, and immune compatibility remains insufficient for long-term outcomes
3	Scaffold complexity	Achieving appropriate mechanical stiffness, conductivity, and ECM mimicry is technically demanding
4	BBB permeability	Limits effective delivery and integration in CNS sites

Recent preclinical innovations like VEGF gradients, electroconductive hydrogels, and CXCL12 chemotactic cues have improved integration, but remain under investigation. Despite significant advances, these biological constraints directly translate to the modest efficacy and limited durability observed in early clinical studies, emphasizing the need for hybrid biomaterial-cell approaches to enhance translation potential (Kelly and Vermeulen, 2017; Chaudhary and Villa-Diaz, 2025; Walkowiak et al., 2025).

### 5.2 Current clinical trials

Several early-phase clinical trials are evaluating NSC and scaffold therapies. Several early-phase clinical trials have investigated NSC-based therapies across a range of neurological conditions. These trials highlight the use of human NSCs derived from some cell sources such as mesenchymal stem cells and induced pluripotent stem cells, and induced pluripotent stem cells in various delivery formats, such as hydrogels and scaffolds. While preliminary outcomes indicate encouraging trends such as improved motor function, reduced infarct size, and slowed disease progression, these effects remain modest and often transient, reflecting the early developmental stage of this field. Moreover, issues such as limited long-term graft survival, suboptimal synaptic integration, and immune compatibility continue to restrict durable functional recovery. These constraints, together with stringent regulatory oversight and heterogeneity in study design, underscore that current findings though promising should be interpreted cautiously until validated by large-scale randomized controlled trials (RCTs).

### 5.3 Regulatory and ethical considerations

Key regulatory and ethical hurdles include:

- Tumorigenesis risk from undifferentiated or genetically modified NSCs.
- Immune rejection, even with autologous cells in inflammatory CNS environments.
- Ethical concerns over fetal-derived or genome-edited stem cells.
- GMP compliance for scaffold fabrication and cell handling.

Despite encouraging early results, challenges such as limited long-term graft survival, immune rejection, and stringent regulatory hurdles continue to restrict clinical scalability. Furthermore, global disparities in regulatory approval timelines and clinical infrastructure have slowed translational momentum. The alignment of RMAT, PRIME, and Japan’s conditional approval systems could accelerate clinical entry of validated NSC therapies.

### 5.4 Market trends and future outlook

**FIGURE 4:**
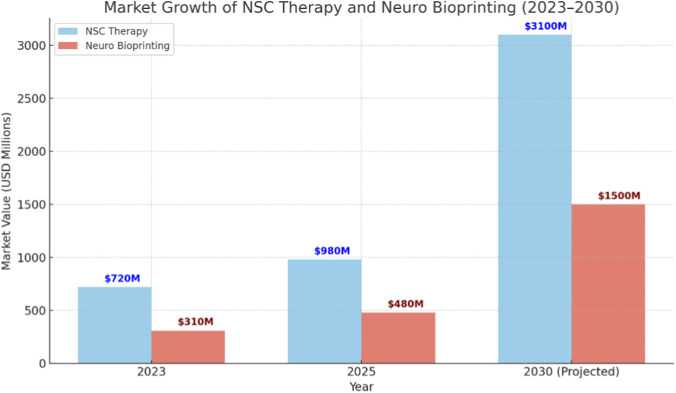
Projected market growth of NSC therapy and neuro bioprinting (2023–2030).

The global market for NSC Therapy and Neuro Bioprinting is exhibiting a robust growth trajectory from 2023 to 2030. Several bioprinting platforms and NSC products are now commercially available, supporting preclinical development and disease modelling (Chaudhary and Villa-Diaz, 2025). As shown in the Figure 4, the NSC therapy market is projected to expand from $720 million in 2023 to $980 million in 2025, reaching approximately $3.1 billion by 2030, driven by a compound annual growth rate (CAGR) of ∼17%. Similarly, the Neuro Bioprinting segment is expected to grow from $310 million in 2023 to $480 million in 2025 (Chaudhary and Villa-Diaz, 2025), reaching $1.5 billion by 2030, with a CAGR of ∼16%. This rapid growth underscores the increasing demand for regenerative therapies and personalized neural repair technologies in treating neurodegenerative diseases and CNS injuries. The rapid market growth reflects not only rising investment but also improved translational infrastructure, including standardized GMP-grade cell production and automated scaffold bioprinting pipelines. Market projections were derived from published industry analyses and are presented to provide contextual insight rather than outcomes from the reviewed studies. However, sustained market momentum depends on resolving long-term graft efficacy, immune safety, and regulatory standardization.

## 6 Roadmap for commercialization and global access

Successful scale-up of NSC-bioprinted therapies into the industry will need academia, industry, and regulators to work together to address scale-up and cost challenges to commercialization. Bridging clinical translation with commercialization depends on establishing GMP-compliant, scalable production pipelines that retain therapeutic efficacy and safety demonstrated in early trials. Today, the production price of patient-specific bioprinted neural scaffolds can extend well over USD 15,000 per product, in a large part due to the GMP-grade cell cultivation, bioink production, and quality control evaluation. Moreover, the absence of harmonized regulatory standards and defined reimbursement pathways further delays market authorization and clinical accessibility. Up to 50 percent production cost savings may be achieved with the automation of bioprinting platforms and through the use of closed-loop bioreactor systems. The most important aspect of Intellectual property (IP) strategy is to recognize the protection of bioink formulations, scaffold designs, and integrated AI algorithms. Equitable access will depend on tiered pricing models, local-scale technology transfer to low- and middle-income countries (LMICs), and adaptive regulatory pathways for diverse healthcare economies. Fair access will mean as well tiered pricing model and technological transfer to the low- and middle-income countries manufacturing centers. Table 10 provides the summary of important points about main commercialization strategies and issues along with possible resolutions.

**TABLE 10:** Commercialization pathways for NSC–bioprinting therapies.

S.No.	Strategy	Challenge	Solution
1	Automated manufacturing	High CAPEX	Public–private partnerships
2	IP protection	Complex multi-component IP	Integrated licensing frameworks
3	Cost reduction	High GMP costs	Modular GMP facilities
4	Global access	Limited LMIC infrastructure	Technology transfer programs
5	Distribution	Surgical workflow integration	Partner with device companies

## 7 Conclusion and future directions

NSCs have emerged as a cornerstone of regenerative neurosurgery, owing to their unique capacity for self-renewal and differentiation into neurons, astrocytes, and oligodendrocytes. This intrinsic plasticity positions NSCs as a promising therapeutic tool for spinal cord injury, stroke, Parkinson’s disease, and traumatic brain injury conditions that account for a significant burden of neurological disability. Unlike conventional symptomatic therapies, NSC-based interventions aim to replace and repair lost neural networks, marking a shift toward true functional regeneration within the central nervous system (CNS).

Recent advances in stem-cell biology and reprogramming technologies have enabled the derivation of patient-specific NSCs from induced pluripotent stem cells (iPSCs) or through direct reprogramming, reducing immune rejection and enabling precision-tailored treatments. Such progress, combined with the refinement of molecular and imaging markers for NSC characterization, has greatly enhanced the reliability of preclinical and early clinical studies.

From a neurosurgical perspective, safe, precise, and reproducible delivery of neural stem cell (NSC)–based therapies remains a principal determinant of clinical success. Achieving therapeutic benefit depends not only on the biological properties of transplanted cells but also on the development of dedicated neurosurgical techniques, instruments, and delivery strategies capable of targeting vulnerable central nervous system (CNS) regions with minimal collateral injury. Current neurosurgical delivery routes under investigation include direct intracerebral or intraparenchymal implantation for focal lesions such as traumatic brain injury or ischemic stroke, intraventricular administration for widespread parenchymal exposure, and intrathecal or intramedullary injection for diffuse spinal cord injury. Each route presents unique technical challenges related to tissue compliance, cerebrospinal fluid dynamics, and graft retention, underscoring the need for procedure-specific neurosurgical optimization.

Importantly, while 3D bioprinting, however, several translational challenges persist: limited graft survival, risk of tumorigenicity, immune rejection, and ethical concerns related to embryonic sources. Furthermore, integrating stem-cell therapies into standard neurosurgical practice requires overcoming technical barriers such as graft vascularization, maintenance of the BBB, and precision placement during surgery. The lack of standardized GMP-compliant protocols, variable clinical outcomes, and incomplete long-term safety data further complicate regulatory approval and widespread adoption. Future directions in this field should focus on three major priorities:

1. Engineering vascularized and BBB-integrated scaffolds to support long-term graft survival and functional integration.
2. Developing standardized, GMP-compliant manufacturing pipelines for hybrid cell–biomaterial constructs to ensure safety and reproducibility.
3. Leveraging AI-driven modelling and digital neurosurgical planning to optimize scaffold design, predict differentiation outcomes, and enhance intraoperative precision.

Realizing these goals will require close collaboration among neurosurgeons, bioengineers, materials scientists, and immunologists, supported by sustained clinical and regulatory partnerships. Several biotechnology firms are already advancing NSC-based therapies for Parkinson’s disease, SCI, and stroke into early-phase clinical trials, reflecting growing commercial confidence in their translational feasibility. 3D neuro-bioprinting and organoid models are further accelerating drug testing and personalized surgical simulation, complementing regenerative efforts *in vivo*.

In summary, the convergence of NSC therapy, 3D bioprinting, and precision neurosurgery is reshaping the therapeutic landscape of neurodegenerative and traumatic CNS disorders. Although considerable challenges remain, coordinated interdisciplinary research and ethical oversight promise to translate these experimental advances into clinically viable, restorative solutions for conditions such as SCI, stroke, Parkinson’s disease, and TBI, ultimately moving neurosurgery from structural repair toward true neural regeneration and functional recovery.
